# Tumor Suppressor DAPK1 Catalyzes Adhesion Assembly on Rigid but Anoikis on Soft Matrices

**DOI:** 10.3389/fcell.2022.959521

**Published:** 2022-07-19

**Authors:** Ruifang Qin, Shay Melamed, Bo Yang, Mayur Saxena, Michael P. Sheetz, Haguy Wolfenson

**Affiliations:** ^1^ Department of Biological Sciences, Columbia University, New York City, NY, United States; ^2^ Department of Genetics and Developmental Biology, Rappaport Faculty of Medicine, Technion—Israel Institute of Technology, Haifa, Israel; ^3^ Mechanobiology Institute, National University of Singapore, Singapore, Singapore; ^4^ Department of Biomedical Engineering, Columbia University, New York City, NY, United States; ^5^ Department of Biochemistry and Molecular Biology, University of Texas Medical Branch, Galveston, TX, United States

**Keywords:** rigidity sensing, integrin adhesions, anoikis, DAPK, cell decision

## Abstract

Cancer cells normally grow on soft surfaces due to impaired mechanosensing of the extracellular matrix rigidity. Upon restoration of proper mechanosensing, cancer cells undergo apoptosis on soft surfaces (anoikis) like most normal cells. However, the link between mechanosensing and activation of anoikis is not clear. Here we show that death associated protein kinase 1 (DAPK1), a tumor suppressor that activates cell death, is directly linked to anoikis activation through rigidity sensing. We find that when rigidity sensing is decreased through inhibition of DAPK1 activity, cells are transformed for growth on soft matrices. Further, DAPK1 catalyzes matrix adhesion assembly and is part of adhesions on rigid surfaces. This pathway involves DAPK1 phosphorylation of tropomyosin1.1, the talin1 head domain, and tyrosine phosphorylation of DAPK1 by Src. On soft surfaces, DAPK1 rapidly dissociates from the adhesion complexes and activates apoptosis as catalyzed by PTPN12 activity and talin1 head. Thus, DAPK1 is important for adhesion assembly on rigid surfaces and the activation of anoikis on soft surfaces through its binding to rigidity-sensing modules.

## Introduction

In the early studies of cancer cells *in vitro*, it was evident that they could grow on widely different substrates, particularly on soft agar, which typically caused apoptosis of normal cells (a process called *anoikis*). This result indicated either that the cancer cells could not sense the surface properly, or that they were stimulated for growth under all conditions through e.g. hyper-activation of receptor tyrosine kinases (RTK), Ras, or other key signaling pathways. Evidence for the former possibility came from recent studies showing that metastatic cancer cells (e.g., MDA-MB-231, HT1080, SKOV3) undergo *anoikis* on soft agar after restoration of rigidity sensing (Yang et al., 2019). This was done through normalization of the expression levels of cytoskeletal proteins involved in rigidity sensing (Ghassemi et al., 2012; Wolfenson et al., 2016). Interestingly, subsequent depletion of other proteins involved in sensing matrix rigidity led to restoration of transformed growth. However, the mechanism by which the rigidity sensing process activated *anoikis* remained unclear.

DAPK1 is a calcium⁄calmodulin-dependent protein kinase with cell death-inducing functions (Cohen et al. 1997; Bialik and Kimchi, 2014). Not only is DAPK1 activity linked to *anoikis* but also it is a tumor suppressor which is depleted in many cancers (Lévy et al., 2004; Raval et al., 2007; Michie et al., 2010). Classically, the activation of DAPK1 is considered to occur by calcium-calmodulin binding, which enables the dephosphorylation of the auto-inhibitory S308 site within DAPK1 (Shohat et al., 2001). There is, however, additional evidence of inhibition through phosphorylation of vicinal tyrosines (Y490/Y491 in human DAPK1) and activation (as observed in immune cells) by tyrosine phosphatase activity (Wang et al., 2007). Some studies have linked DAPK1 to cytoskeletal proteins and motility, including the ability of DAPK1 to phosphorylate tropomyosin1 [Tpm1.1; formerly known as Tm skα (Geeves et al., 2015)] and myosin light chain (Bialik et al., 2004; Houle et al., 2007). Notably, Tpm1 expression is sufficient to restore *anoikis* in metastatic cancer cells (Wang et al., 2019). Further, DAPK1 has a role in cell polarization and migration through an interaction with the talin1 head domain (Kuo et al., 2006), suggesting a potential role for DAPK1 in regulation of mechanosensing. Still, no direct link between DAPK1 activity, rigidity sensing, and *anoikis* has been established.

Early rigidity sensing in differentiated fibroblasts involves formation of relatively complicated protein modules that are capable of transducing matrix rigidity into biochemical signals to either activate growth on rigid matrices or apoptosis on soft matrices. The rigidity sensors are non-muscle sarcomere-like units of about 2 μm in length, with anti-parallel actin filaments anchored to the matrix adhesions and a bipolar myosin IIA filament in the middle (Wolfenson et al., 2019). To test the rigidity, these units contract the matrix to a constant distance of about 100 nm in total, and the force which is developed is sensed. The receptor tyrosine kinases, AXL and ROR2, regulate the displacement and the duration of the contractions, respectively (Yang et al., 2016). EGFR and HER2 can activate contractions on rigid but not on soft matrices downstream of Src phosphorylation (Saxena et al., 2017a). Surprisingly, normal rigidity sensing involves force-dependent cleavage of talin1 by calpain that catalyzes growth on rigid surfaces through the generation of free talin1 rod domains (Saxena et al., 2017b). The velocity of the contraction depends upon Tpm2.1 binding to the actin filaments, and AXL phosphorylation of Tpm2.1 on Y214 is needed for adhesion formation (Yang et al., 2016). Perhaps because this is a complicated sensory module, depletion of this module’s activity follows upon the depletion of any one of many cytoskeletal proteins, including Tpm2.1, myosin IIA, and α-actinin4 (Meacci et al., 2016; Wolfenson et al., 2016). Loss of rigidity sensing activity, however, enables cell growth on soft surfaces, as is the case with many cancers, whereas proper formation of such modules leads to apoptosis on soft surfaces, which begs the question of how rigidity sensing activates apoptosis.

Here we studied the activation of *anoikis* through rigidity sensors and found that DAPK1 activated *anoikis* and that inhibition of DAPK1 was sufficient to enable growth on soft surfaces. Under control conditions, assembly of rigidity sensors and adhesion formation normally involved DAPK1 activity and particularly required the phosphorylation of Tpm1.1. On rigid substrates, DAPK1 associated with adhesions in a Tpm1.1-dependent process that involved Tpm1.1 phosphorylation by DAPK1. On soft substrates, DAPK1 rapidly dissociated from the adhesion complexes and activated apoptosis. Apoptosis was catalyzed by the constitutively phosphorylated mutant of Tpm1.1 and talin1 cleavage, but it was inhibited by Src and EGFR activity. DAPK1 assembly in adhesions depended on Y490/Y491 phosphorylation by Src or EGFR, and tyrosine (Y) to phenylalanine (F) mutations catalyzed apoptosis. Inhibition of the tyrosine phosphatase, PTPN12, inhibited *anoikis* potentially by blocking DAPK1 activation. Thus, we suggest that DAPK1 is an important component in the assembly of rigidity sensors and adhesions through interactions with Tpm1.1 and the talin1 head, leading to growth on rigid matrices and *anoikis* on soft ones.

## Materials and Methods

### Cell Culture

Mouse embryonic fibroblast cells (MEFs) were generated by J. Sap’s laboratory (Su et al., 1999). MDA-MB-231 and MCF10A cells were obtained from J. Groves (University of California, United States and Mechanobiology Institute, National University of Singapore, Singapore). The talin1^−/−^ mouse fibroblast cell line dj26.28 was generated and maintained as described previously (Priddle et al., 1998). The MEFs and MDA-MB-231 cells were cultured in Dulbecco’s Modified Eagle Medium (DMEM) (Thermo Fisher Scientific) supplemented with 10% fetal bovine serum (FBS) (Atlanta Biologicals) and 100 IU/ml Penicillin-Streptomycin (Sigma) at 37°C and 5% CO_2_. MCF10A cells were cultured at 37°C in a 5% CO_2_ incubator in DMEM (Thermo Fisher Scientific) supplemented with 20 ng/ml EGF (Thermo Fisher Scientific), 10 ng/ml bovine insulin (Sigma), 500 ng/ml hydrocortisone, 5% horse serum albumin (Thermo Fisher Scientific), and 100 IU/ml Penicillin-Streptomycin (Sigma). Imaging experiments were conducted using starvation medium without phenol red or in Ringer’s buffer (150 mM NaCl, 5 mM KCl, 1 mM CaCl_2_, 1 mM MgCl_2_, 20 mM Hepes, and 2 g/L glucose, pH 7.4). Pharmacological inhibitors were as follows: DAPK1 inhibitor (100 nM, EMD Millipore), EGFR inhibitor Gefitinib (10 nM, Santa Cruz), PP2 (200 nM, abcam), calpain Inhibitor ALLN (100 μM, Santa Cruz), PTPN12 inhibitor (1.5 μM, EMD Millipore). Cells were trypsinized using TrypLE (Thermo Fisher Scientific) on the following day and suspended in Ringer’s buffer at 37°C for 30 min prior to plating on human plasma fibronectin (10 μg/ml, Roche) coated glass-bottom dishes (MatTek) or pillars. The 0.2 kPa gels used for testing cells’ growth on soft matrices were purchased from Soft Substrates^TM^.

### Transfection and Plasmids

Transfections were carried out 1 day before measurements using Lipofectamine Plus Reagent (Invitrogen) according to the manufacturer’s instructions. Expression vectors encoding the following fluorescent fusion proteins were used: EGFP-DAPK1 WT, EGFP-DAPK1 K42A, mCherry-DAPK1 WT, mCherry-DAPK1 K42A, Emerald-Tpm1.1 WT, Emerald-Tpm1.1 S283E (phosphomimetic mutant), Emerald-Tpm1.1 S283A (non-phosphorylated mutant), mCherry-paxillin, GFP-talin1 WT, mRuby-talin1 head, mCherry-talin1 rod, GFP-non-cleavable talin1, EGFP-DAPK1-DYD (phosphomimetic mutant), EGFP-DAPK1-DYF (non-phosphorylated mutant), EGFP-DAPK1-DYF-K42A (non-phosphorylated inactive mutant), mCherry-PTPN12. MEFs were seeded into a 6-well dish on day 0 and transfected with control shRNA (Sigma) or mouse DAPK1 shRNA (Sigma) using Lipofectamine Plus Reagent (Invitrogen) on day 1, followed by selection in 2 μg/ml puromycin for 72 h. Control siRNA, mouse Src siRNA and mouse PTPN12 siRNA (Santa Cruz) were transfected using siRNA transfection reagent (Santa Cruz).

### Apoptosis Assays

Apoptotic cells were identified for appearance of apoptotic morphology including membrane blebbing and cell rounding. Live cells retained their normal spread morphology. Apoptotic cells were also determined by anti-Annexin V immunostaining. The Life Technologies™ Click-iT® Plus TUNEL assay with Alexa Fluor® 647 dye was used to detect the fragmented DNA.

### Pillar Fabrication

Fabrication of pillar arrays with diameters of 500 nm was performed as previously reported (Ghassemi et al., 2012). Polydimethylsiloxane (PDMS, Sylgard 184, Dow Corning) was mixed thoroughly with its curing agent (10:1) for at least 5 min, centrifuged (2000 RPM, 3 min, room temperature) and degassed in a vacuum for about 10 min. A drop of PDMS was then placed on the top of the Poly (methyl methacrylate) (PMMA) mold and degassed again in a vacuum for a few minutes. At the same time, a glass-bottom Petri dish (No.0 Coverslip, MatTek) was treated with O_2_ plasma for 1 min in order to increase adhesion of the PDMS to the glass after curing. The mold with PDMS was then inverted and placed onto the bottom of the Petri dish, and a 1 g weight was placed on the top of mold to make the PDMS thinner. The PDMS was then cured at 70°C for 12–14 h in order to achieve a Young’s modulus of 2 ± 0.1 MPa. The mold was then peeled off the PDMS while immersed in ethanol. Individual pillars were 500 nm in diameter with a center-to-center distance of 1 μm.

For the dual stiffness pillar fabrication, Coumarin 343 (Sigma-Aldrich), which bleaches under UV treatment, was premixed in PDMS for the marking of areas with different rigidity. The dyed PDMS was applied to the master molds, which were spun at 6600 RPM for 3 s to ensure even coating. After the PDMS was cured at 70°C for 12–14 h, it was carefully peeled off the molds and placed (pillar-side up) onto the O_2_ plasma-treated glass-bottom Petri dish. Prior to UV/Ozone treatment, nickel TEM grids (SPI supplies) were laid on the pillar tops. Pillar substrates covered by grids were then placed in the UV/Ozone chamber (Bioforce Nanosciences UV/Ozone ProCleaner Plus) for 2 h.

### Traction Force Measurements

Cells were spreading on pillar arrays coated with fibronectin (10 μg/ml, Roche). Time lapse imaging of pillars was performed with bright-field microscopy using an Orca-flash 2.8 camera (Hamamatsu) attached to an inverted microscope (Olympus IX81) maintained at 37°C running MicroManager software (Edelstein et al., 2010). Images were recorded at 1 Hz using a ×100 objective (1.4 NA oil immersion, Olympus). Videos were processed with ImageJ (National Institutes of Health) using the Nano Tracking plugin to track the position of pillars. The time-series positions of all pillars in contact with the cell were fed into a MatLab program (MathWorks) to generate displacement maps as explained previously (Wolfenson et al., 2016; Saxena et al., 2017a).

### Immunostaining and Fluorescence Microscopy

Cells were fixed for 15 min with 4% formaldehyde solution (Sigma, diluted from 37% in PBS). Fixed cells were then permeabilized with 0.2% Triton X-100 in PBS (V/V, Sigma) for 5 min, and blocked in 2% bovine serum albumin (BSA) in PBS (W/V, Sigma) at room temperature for 1 hour. Primary antibody was diluted in 1% BSA and incubated with samples overnight at 4°C. Secondary antibody was diluted in 1% BSA and incubated with samples for 1 h at room temperature. For immunostaining, the following primary antibodies were used: anti-paxillin (Abcam, dilution 1:250), anti-DAPK1 (BD Biosciences, dilution 1:50), anti-pSer308 DAPK1 (LSBio, dilution 1:100), anti-Annexin V (Abcam, dilution 1:500), anti-PTPN12 (Bethyl Laboratories, Inc., dilution 1:200). Secondary antibodies were Alexa Fluor 488 goat anti-mouse IgG (H+L), Alexa Fluor 555 goat anti-rabbit IgG (H+L) and Alexa Fluor 647 goat anti-rabbit IgG (H+L) (Thermo Fisher Scientific).

Total internal reflection fluorescence (TIRF) images were taken using an Olympus IX81 fluorescence microscope with a ×60, 1.45 NA oil-immersion objective and a Cool Snap FX cooled CCD camera (Photometrics) controlled by MicroManager software (Edelstein et al., 2010). Confocal microscopy was performed on a Zeiss LSM 700 laser-scanning confocal microscope, using a ×63/1.4 NA oil-immersion objective. Quantification of images was performed with ImageJ (National Institutes of Health).

For the fluorescence recovery after photobleaching (FRAP) experiment, MEFs transfected with EGFP-DAPK1 K42A or EGFP-DAPK1 WT were allowed to spread on fibronectin-coated pillars for 20 min and then focal adhesion areas were bleached using “fast bleach” on Zeiss LSM 700. Fluorescence intensity recovery was simultaneously recorded at 1 frame per 2 s. The images were then corrected for background using ImageJ (National Institutes of Health). Single exponentials were then fitted to the recovery profiles in Excel and *SigmaPlot 12.0* software (Systat Software Inc.) to calculate half-times (t_1/2_).

### Statistical Analysis

Statistical analysis and graph plotting were performed using *SigmaPlot 12.0* software (Systat Software Inc.) and Matlab (Math Works). Two-tailed Student’s t-test was performed when two cases were compared. One-way-ANOVA was performed for multi-group comparison. *p* values < 0.05 were considered statistically significant.

## Results

### Inhibition of Death Associated Protein Kinase 1 Contributes to Cell Growth on Soft Matrices

**FIGURE 1:**
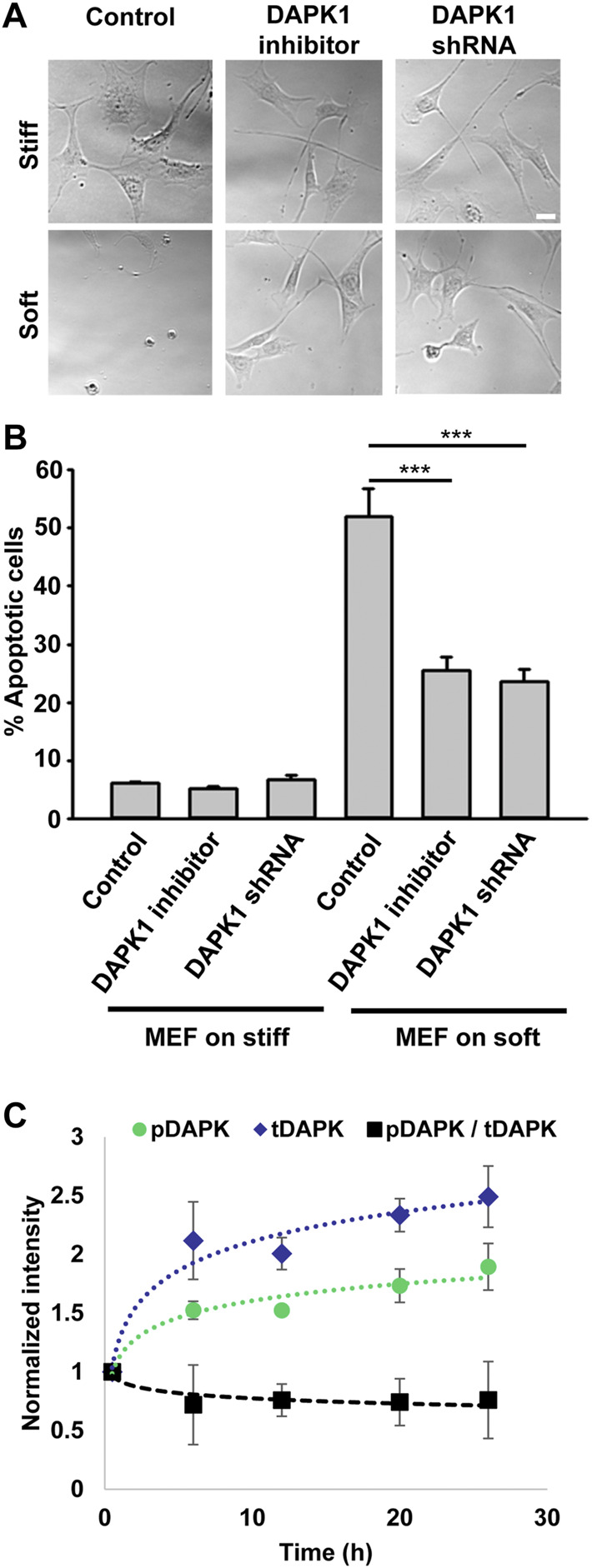
Inhibition of DAPK1 contributes to cell growth on soft matrices. **(A)** Bright field images of MEFs with DAPK1 inhibitor or stable DAPK1 shRNA MEFs on stiff or soft surfaces. Scale bar: 25 μm. **(B)** MEF cells with DAPK1 inhibitor or stable DAPK1 shRNA MEFs were incubated in culture for 1 day on soft or stiff surfaces. Apoptotic cells were identified for appearance of apoptotic morphology including membrane blebbing and cell rounding. Live cells retained their normal spread morphology. Graphs represent means ± SEM of at least two independent experiments. ***p < 0.001. **(C)** MEF cells were plated on 0.2 kPa gels and the levels of pS308 DAPK1 (pDAPK) and total DAPK1 (tDAPK) were monitored by double immunostaining of the cells over time. Graphs represent means ± SD. Experiment was repeated twice. ***p < 0.001.

Because DAPK1 activity correlated with *anoikis*, autophagy, and TNF-alpha-induced apoptosis (Cohen et al., 1999; Inbal et al., 2002; Wang et al., 2002), we wanted to test if it was responsible for apoptosis of cells on soft substrates. As shown in Figure 1A, mouse embryonic fibroblasts (MEFs) on soft substrates (0.2 kPa) activated apoptosis with the characteristic changes in morphology, including membrane blebbing and cell rounding. Apoptosis of MEFs on soft substrates was blocked by using the DAPK1 inhibitor, (4Z)-4-(3-Pyridylmethylene)-2-styryl-oxazol-5-one, or in stable DAPK1 shRNA MEFs (Figures 1A,B; Supplementary Figure S1A). Similar results were observed in MCF10A, normal human epithelial cells (Supplementary Figure S1B). Further quantitative analysis of cellular apoptosis based on Annexin V immunostaining or Tunel assay matched cell apoptotic morphology results (Supplementary Figure S1C). Thus, in both fibroblasts and epithelial cells, the cells were protected from apoptosis on soft matrices by inhibition of DAPK1.

The autophosphorylation of DAPK1 at S308 was found to be the major mechanism of DAPK1 inhibition in cells and that site was exposed for dephosphorylation upon binding of calcium/calmodulin (Shohat et al., 2001). To test the involvement of S308 in *anoikis*, we plated cells on 0.2 kPa surfaces and performed double immunostaining at different time points over 24 h for phosphorylated DAPK1 S308 and total DAPK1. At the same time, we stained DAPK1 shRNA cells to confirm specificity (Supplementary Figure S1D). This analysis showed an increase in the level of total DAPK1 within the first 6 h, and a less prominent increase in the level of pS308 phosphorylation in the same cells, evident by the decrease in the ratio of phosphorylated DAPK1 S308/total DAPK1 over time (Figure 1C). In contrast, both pS308 and total DAPK1 levels in DAPK1 shRNA cells were low and did not change over time (Supplementary Figure S1D). Thus, soft matrices lead to enhanced DAPK1 expression and overall decreased DAPK1 inhibition (unphosphorylated S308).

### Death Associated Protein Kinase 1 Regulates Adhesion Growth and Force Production

**FIGURE 2:**
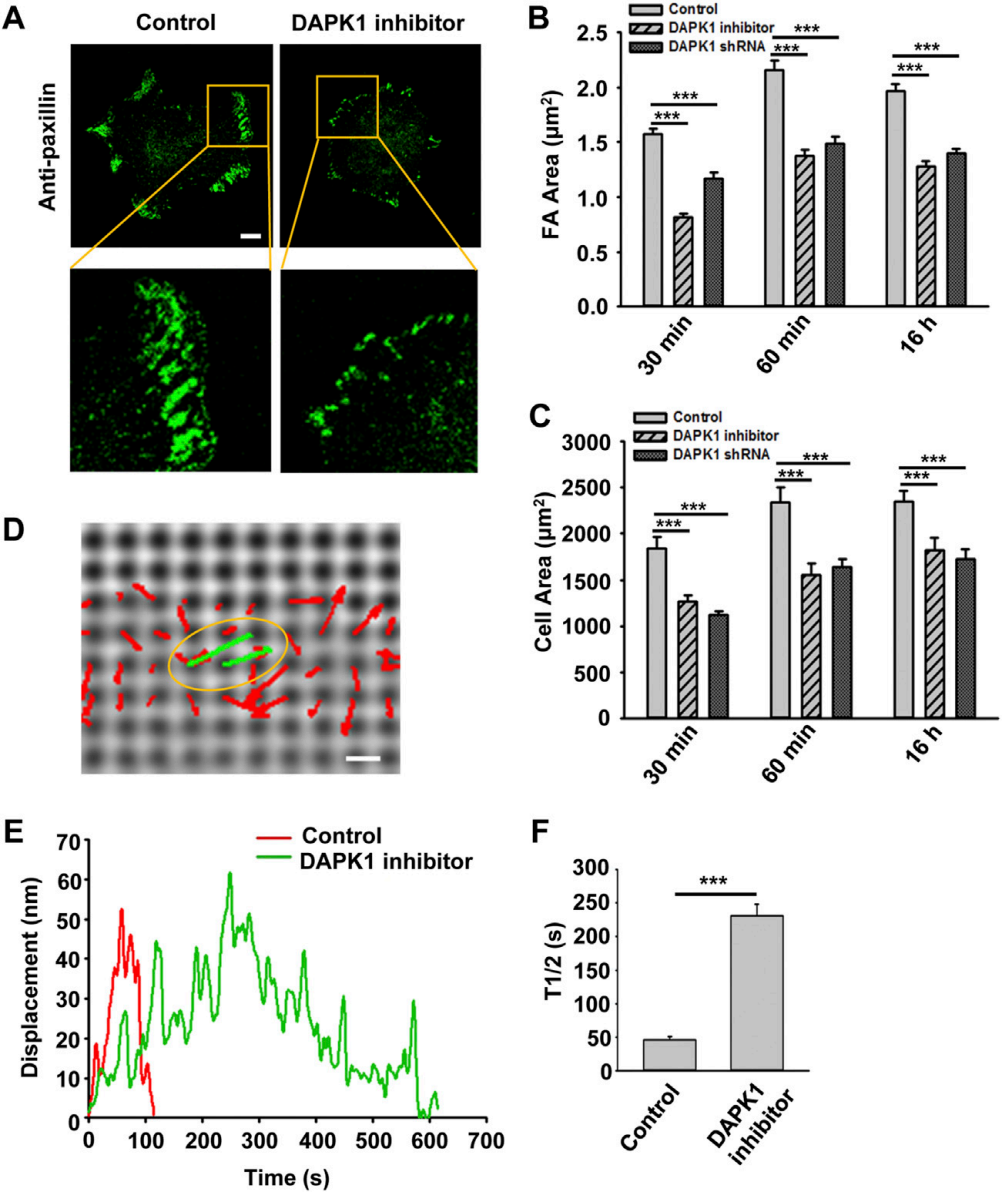
DAPK1 regulates adhesion growth and force production. MEFs treated with DAPK1 inhibitor or stable DAPK1 shRNA MEFs were fixed after spreading on fibronectin-coated glass dishes for 30 min, 60 min or 16 h, followed by anti-paxillin/AlexaFluor 488 immunostaining. **(A)** Micrographs showing the distribution of paxillin in cells fixed after spreading on fibronectin-coated glass for 30 min. Scale bar: 10 μm. **(B)** Quantification of the adhesion sizes (n > 400 adhesions from >10 cells in each case, mean ± SEM). ***p < 0.001. **(C)** Average area ± s.e.m of the cells (n > 10 cells in each case). ***p < 0.001. Experiment was repeated three times. **(D)** Displacements (red arrows) of pillars that show the contractile pair (green arrows marked with yellow oval) at the periphery of a spreading MFF during phase 2 of cell spreading (Dobereiner et al., 2004). Scale bar: 1 μm. **(E)** Displacements versus time of contractile pillars in MEFs treated with or without DAPK1 inhibitor. **(F)** Mean ± SEM of the T1/2 (time of contraction above half-maximal displacement) distributions of pillar displacements by MEFs during phase 2 of cell spreading before and after treatment with DAPK1 inhibitor. ***p < 0.001. N > 30 pillars in each case. Experiment was repeated twice.

Next, we wished to test the involvement of DAPK1 in cell adhesion. Inhibition of DAPK1 activity or DAPK1 shRNA both slowed cell spreading, and particularly inhibited formation of mature focal adhesions (Figures 2A–C). Careful examination of cell spreading videos indicated that the early spreading phase (approximately first 10 min of spreading) was nearly normal without DAPK1 activity, but the later spreading phase that was associated with rigidity-sensing and contractile activity [phase 2 of spreading (Dobereiner et al., 2004)] was inhibited in correlation with decreased adhesion size. Thus, it seemed that DAPK1 activity was needed to form mature adhesions.

We previously showed that local rigidity-sensing contractions through transient contractile units correlated with adhesion formation and maturation (Ghassemi et al., 2012; Wolfenson et al., 2016). To test if DAPK1 inhibition altered adhesion formation by affecting rigidity sensing contractions, cells were plated on fibronectin-coated 500 nm diameter polydimethylsiloxane (PDMS) pillars. When cells reached phase 2 of spreading, they contracted pairs of pillars toward each other (Figure 2D) to a peak distance of 50–60 nm each for about 30 s and then relaxed the pillars at about the same velocity (2–3 nm/s) (Figure 2E), consistent with previous studies (Ghassemi et al., 2012; Wolfenson et al., 2016). Inhibition of DAPK1 caused a dramatic decrease in the velocity of pulling (0.3–0.5 nm/s when measuring from initiation of pulling to peak displacement) with repeated release events (Figures 2E,F), but did not alter the maximum pillar displacement (∼60 nm) or CU density (Supplementary Figure S1E). Thus, the decrease in adhesion formation after DAPK1 inhibition correlated with a decrease in the rate of rigidity-sensing contractions. Further, DAPK1 inhibition resulted in small adhesions that appeared similar to those of transformed cells rather than adhesions of normal fibroblasts (Figure 2A).

### Death Associated Protein Kinase 1 Co-localizes With Tpm1 and Targets to Focal Adhesion Sites

**FIGURE 3:**
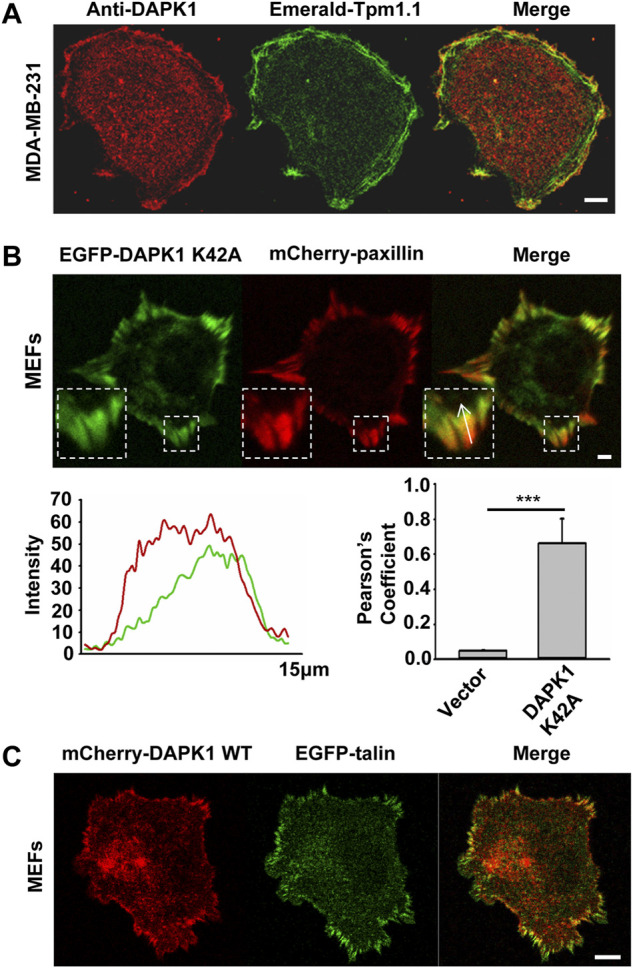
DAPK1 co-localizes with tropomyosin1 and targets to focal adhesion sites. **(A)** MDA-MB-231 cells were transfected with Emerald-Tpm1.1 WT. Cells were further analyzed by anti-DAPK1/AlexaFluor555 immunostaining. Scale bar: 10 μm. **(B)** Total internal reflection fluorescence (TIRF) image of MEF co-transfected with EGFP-DAPK1 K42A and mCherry-paxillin after 30 min spreading on fibronectin-coated glass dish. Scale bar: 10 μm. Fluorescence intensity line profiles are from the area of the representative adhesions covered by the white arrow in the merged images. Pearson coefficients with Image J were calculated to determine the colocalization of DAPK1 to paxillin-stained adhesions. Data are means ± SEM for 10 individual cells. ***p < 0.001. **(C)** Representative images of MEFs co-transfected with EGFP-talin and mCherry-DAPK1 WT that were fixed after 30 min of spreading on fibronectin-coated glass dish. Scale bar: 10 μm.

Because the rigidity sensing contractions depended upon DAPK1 and because DAPK1 can phosphorylate Tpm1.1 (Houle et al., 2007), we next analyzed the distribution of DAPK1 and Tpm1.1 in early spreading cells. In Tpm1.1-transfected MDA-MB-231 cells (human breast cancer cell line) which normally lack endogenous Tpm1 (Houle et al., 2007), DAPK1 overlapped with Tpm1.1 predominantly at the cell edge (Figure 3A), where rigidity sensing activity typically occurs.

To dynamically track DAPK1 in cells without causing cell blebbing and apoptosis due to its overexpression, we next used an EGFP-DAPK1 K42A construct [the inactive form of DAPK1 (Chen et al., 2005)] in MEFs. During early spreading of MEFs on fibronectin-coated glass, EGFP-DAPK1 K42A showed significant overlap with paxillin in focal adhesion sites, although the majority of DAPK1 was in the older portion of the adhesions (the proximal edge of the adhesion, closer to the nucleus) (Figure 3B). We further confirmed in static images that DAPK1 WT (in the few cells that did not die) localized to focal adhesions like DAPK1 K42A (Figure 3C; Supplementary Figure S2A). Similar results were observed on fibronectin-coated pillars with either the DAPK1 construct or anti-DAPK1 antibody (Supplementary Figures S2B,C). These results indicated that DAPK1 was part of adhesions on fibronectin-coated surfaces in cells that were rigidity-dependent for growth.

### The Phosphorylation of Tpm1.1 by Death Associated Protein Kinase 1 is Important for Adhesion Maturation but Increases Sensitivity to *Anoikis*

**FIGURE 4:**
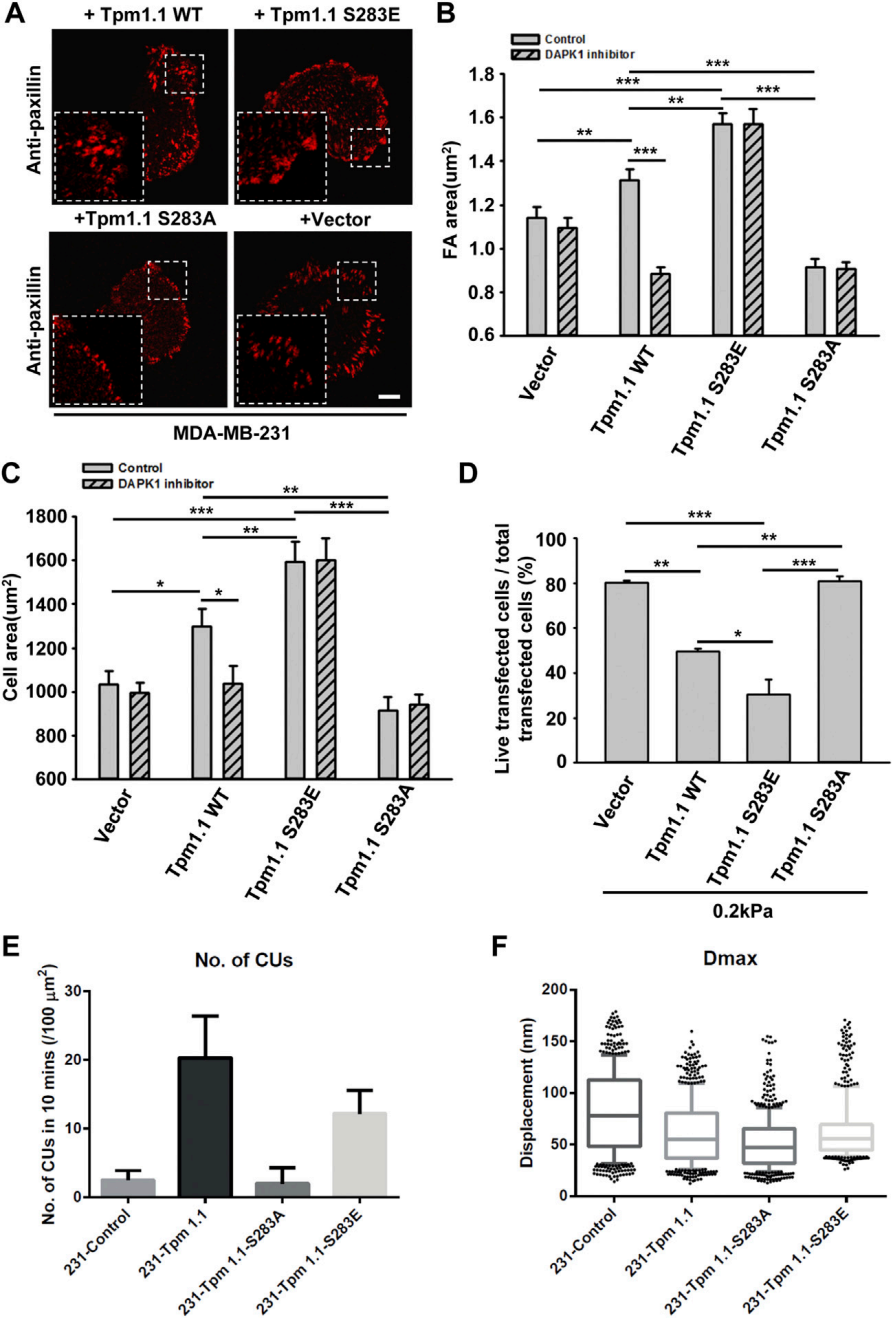
The phosphorylation of Tpm1 by DAPK1 is important for adhesion formation but facilitates *anoikis* on soft substrates. MDA-MB-231 cells were transfected with Emerald-Tpm1.1 WT, Emerald-Tpm1.1 S283E (phosphomimetic mutant), Emerald-Tpm1.1 S283A (non-phosphorylated mutant), or control vector. Cells were spreading on fibronectin-coated glass dishes with/without DAPK1 inhibitor for 30 min, followed by anti-paxillin/AlexaFluor 555 immunostaining. **(A)** Micrographs show the distribution of paxillin. Scale bar: 10 μm. **(B)** Quantification of the adhesion sizes (n > 400 adhesions from >10 cells in each case, mean ± SEM), **p < 0.01, ***p < 0.001. **(C)** Average area ± s.e.m of transfected cells in each group (n > 10 cells in each case). *p < 0.05, **p < 0.01, ***p < 0.001. **(D)** MDA-MB-231 cells were transfected with Emerald-Tpm1.1 WT, Emerald-Tpm1.1 S283E, Emerald-Tpm1.1 S283A or Vector respectively for 1 day and replated on fibronectin-coated 0.2 kPa gels for 1 day. The percentage of transfected cells that were still alive was quantified. The mean ± SEM of at least two independent experiments is described. For each experiment, 100–150 cells were analyzed for each transfection point. *p < 0.05, **p < 0.01, ***p < 0.001. **(E)** Average number of CUs/100 μm^2^ generated by different transfected cells on pillars per 10 min. **(F)** Box and whiskers plots of pillar maximum displacement summary from different transfected cells.

Since DAPK1 was found to phosphorylate Tpm1.1 on S283 (Houle et al., 2007), we postulated that the inhibition of DAPK1 activity may have altered cell adhesion formation through the inhibition of Tpm1.1 phosphorylation. To test this possibility, we transfected MDA-MB-231 cells with mutant forms of Tpm1.1 which were blocked for phosphorylation, Tpm1.1 S283A, or mimicked the phosphorylated state, Tpm1.1 S283E. We tracked adhesions after immunostaining for paxillin and found that the S283A mutant led to formation of small adhesions that were similar in size to those in non-transfected MDA-MB-231 cells. In contrast, with the Tpm1.1 S283E mutant, larger adhesions formed, and cells spread to larger areas, similar to Tpm1.1 WT (Figures 4A–C). Notably, the Tpm1.1 S283E phosphomimetic mutant conferred resistance to the DAPK1 inhibitor (Figures 4B,C), providing evidence for the specificity of the inhibitor. Further, whereas transfection of Tpm1.1 WT, and more so of Tpm1.1 S283E, induced apoptosis of MDA-MB-231 cells on soft surfaces, Tpm1.1 S283A-transfected cells displayed resistance to apoptosis on soft surfaces (Figure 4D; Supplementary Figure S2D). In addition, the density of contractile units increased dramatically in the presence of Tpm1.1 and Tpm1.1 S283E in MDA-MB-231 cells (Figure 4E). Further, forces produced by these cells were significantly lower than MDA-MB-231 control cells (Figure 4F), an effect that was similar to restoration of Tpm2.1 expression in these cells (Wolfenson et al., 2016; Yang et al., 2019). Thus, the expression of Tpm1.1 and its phosphorylation at the DAPK1 phosphorylation site was important for formation of mechanosensing contractile units, which was accompanied by adhesion maturation in MDA-MB-231 cells but facilitated *anoikis* on soft substrates.

### Talin1 Head Increases Death Associated Protein Kinase 1 Localization to Adhesions and Facilitates Apoptosis

**FIGURE 5:**
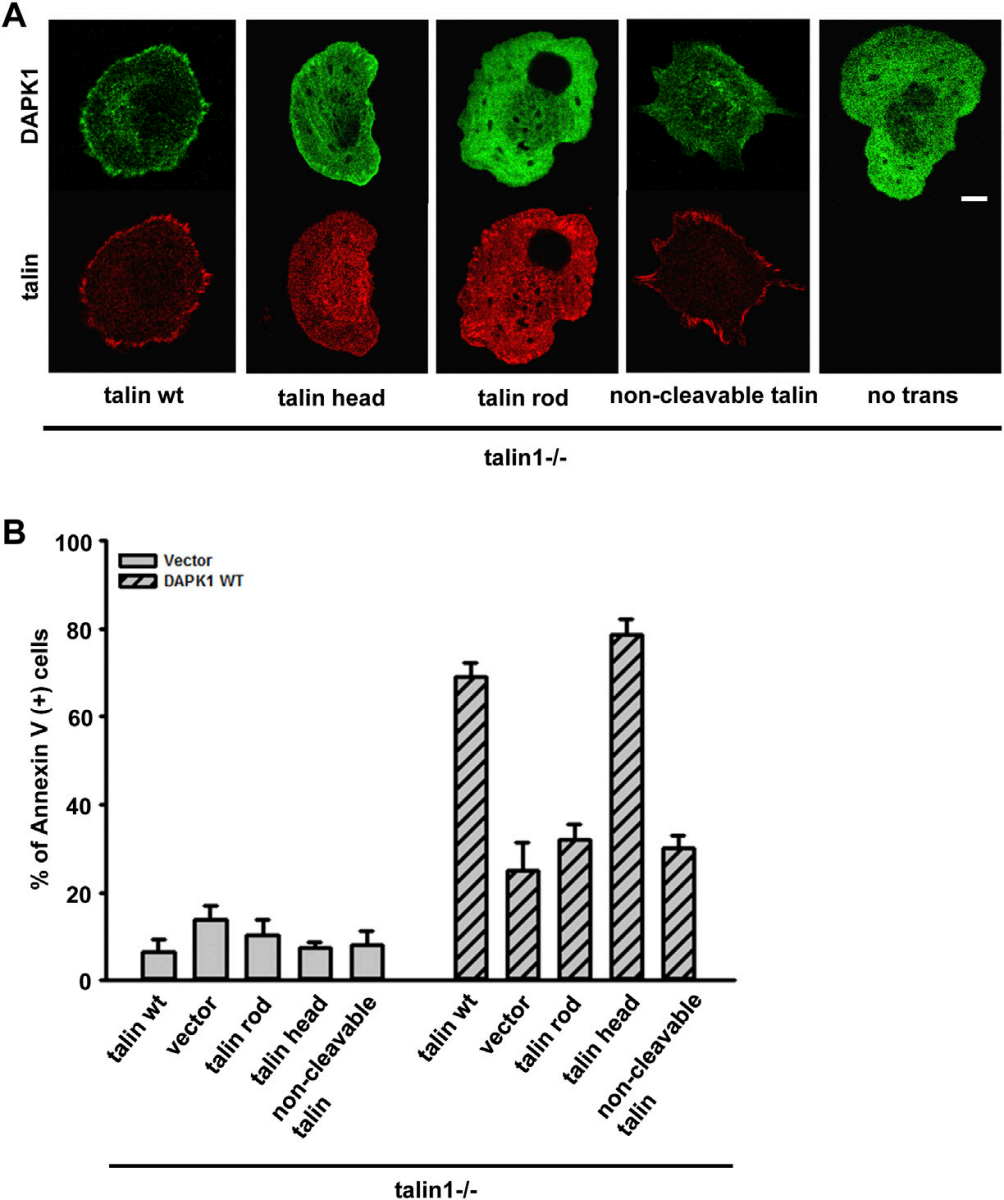
Talin1 head aids in the localization of DAPK1 to the adhesions and facilitates apoptosis. **(A)** Talin1^−/−^ cells were co-transfected with DAPK1 and talin1 constructs (talin1 WT, talin1 head, talin1 rod, non-cleavable talin1). Cells were spread on fibronectin-coated glass dishes for 30 min. Scale bar: 10 μm. **(B)** Talin1^−/−^ cells were co-transfected with DAPK1 WT and talin1 constructs (talin1 WT, talin1 head, talin1 rod, NC talin1) for 1 day, followed by anti-Annexin V immunostaining. The percentage of Annexin V positive cells in transfected cells was quantified. The mean ± SEM of at least two independent experiments is described. For each experiment, 100–150 cells were analyzed for each transfection point.

Previous studies reported that DAPK1 interacted with talin1 through the head domain (Kuo et al., 2006). In a separate study, we also showed that the assembly of adhesions depended upon talin1 cleavage by calpain, and that cell growth depended upon the presence of the talin1 rod even in the absence of the head (Saxena et al., 2017b). To test if talin1 cleavage was needed for DAPK1 assembly into adhesions, we transfected non-cleavable (NC) talin1, WT talin1 and the rod and head fragments separately in talin1^−/−^ fibroblasts. With WT talin1 and the talin1 head, we found that DAPK1 assembled into focal adhesions; however, neither the talin1 rod nor NC talin1 supported DAPK1 recruitment to adhesions (Figure 5A). Thus, although the rod was needed for growth, the head fragment was needed for DAPK1 assembly in adhesions, which supported the previous findings of an interaction of DAPK1 with the talin head (Kuo et al., 2006). In DAPK1 WT transfected talin1^−/−^ cells with talin1 constructs or MEFs treated with a calpain inhibitor (to prevent talin cleavage), talin1 head and talin1 cleavage caused an increase in DAPK1 apoptotic activity (Figure 5B; Supplementary Figure S3B). When talin1^−/−^ cells were cultured on soft surfaces, they were protected against apoptosis whereas with the WT talin1 they underwent apoptosis. Rescue of talin1^−/−^ cells with NC talin1 did not restore apoptosis on soft surfaces but expression of the talin1 head domain caused greater apoptosis on soft surfaces than did WT talin1 (Supplementary Figures S3A,C). Thus, the cleavage of talin1 by calpain produced the talin1 head domain fragment that increased DAPK1 assembly in focal adhesions and catalyzed apoptosis on soft surfaces.

### Death Associated Protein Kinase 1 is Recruited to Nascent Adhesions, but Rapidly Dissociates When They Form on a Soft Matrix

**FIGURE 6:**
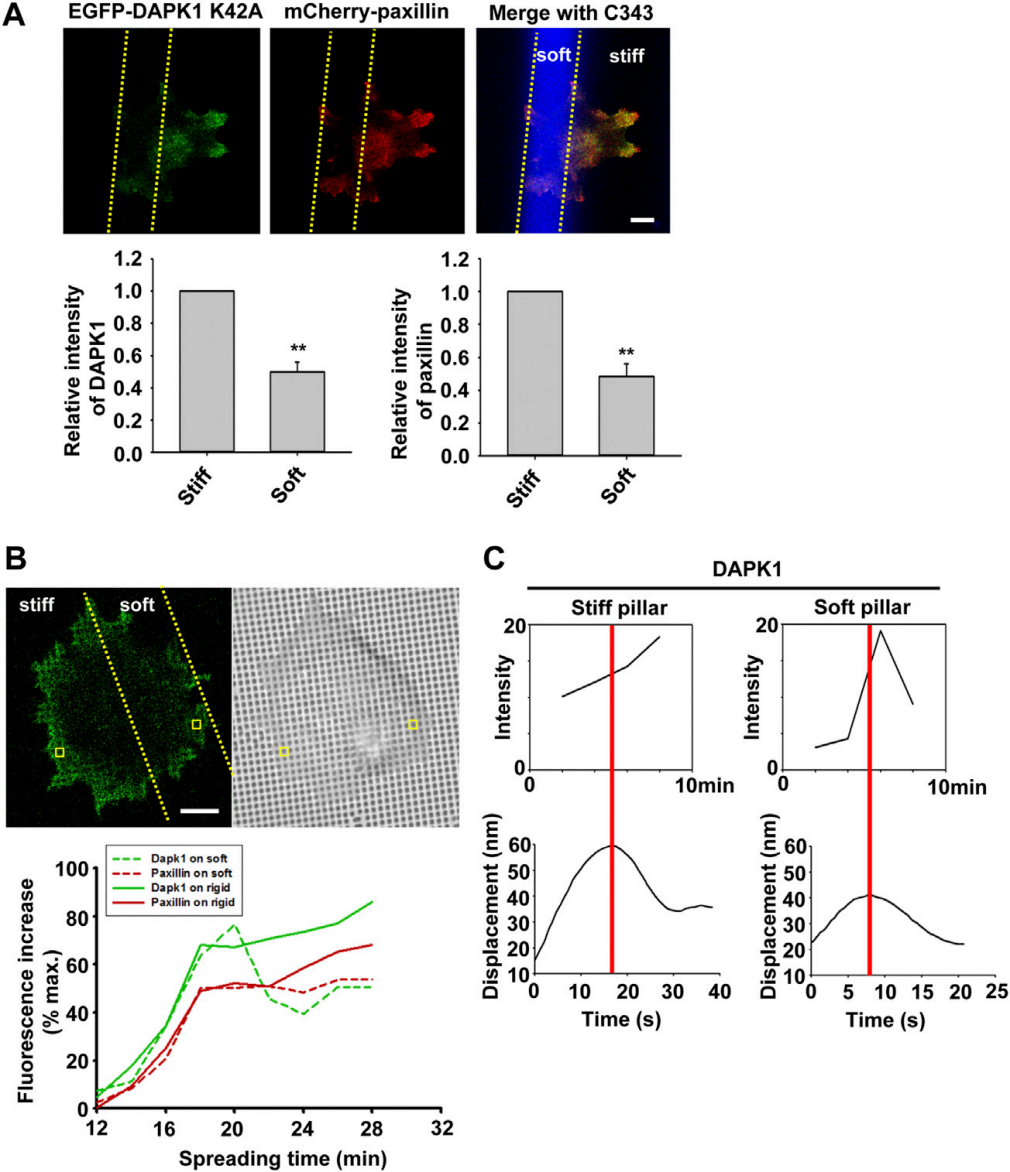
Dynamics of DAPK1 during cell spreading on dual stiffness pillars. **(A)** MEFs were co-transfected with EGFP-DAPK1 K42A and mCherry-paxillin. Cells were spread (30 min) on dual stiffness pillars (9 kPa (Coumarin 343) and 170 kPa), Scale bar: 10 μm. The leading edge protein relative intensity was normalized by dividing the average intensity value within 3 μm of the cell edge in stiff region or soft region by the average intensity value of the cell edge in stiff region of each cell (stiff/stiff or soft/stiff). The relative intensity of DAPK1 or paxillin is presented in the lower panels. **p < 0.01. N = at least 5 different cells in each group. Graphs represent mean ± SEM. **(B)** Upper panel: confocal image showing a cell transfected with EGFP-DAPK1 K42A and mCherry-paxillin on dual stiffness pillars. Scale bar: 10 μm. Lower panel: one adhesion site of the cell corresponding to a single pillar within 2 μm of outward curving cell edges in a stiff or soft region was tracked during cell spreading. The tracked pillar is marked by a yellow box. The relative fluorescence of both molecules was plotted. **(C)** The time course of recruitment of DAPK1 was measured during pillar contraction and relaxation. Displacements versus time of contractile pillars are shown in the lower panel. The peak of the pillar contraction force is marked by a red line.

To confirm that DAPK1 recruitment to adhesions was dependent upon local rigidity, we prepared pillar substrates that had sub-cellular regions with a 20-fold difference in pillar rigidity by exposing PDMS pillars to UV light in localized regions (Song et al., 2008). When MEF cells transfected with EGFP-DAPK1 K42A and mCherry-paxillin were spread on these dual-stiffness pillars for 30 min, both DAPK1 and paxillin staining were lower on the soft pillars compared to the rigid ones (Figure 6A). In time-lapse studies of DAPK1 recruitment to pillars, EGFP-DAPK1 K42A accumulated equally rapidly on soft and stiff pillars at early times, but was then released from the soft pillars whereas it remained associated with the rigid pillars (Figure 6B). We then followed single pillars during the contraction and relaxation to correlate the recruitment level of DAPK1 with contractile unit force. After the peak of contraction force, DAPK1 intensity on the soft pillars decreased rapidly, in sharp contrast to its increase in intensity on stiff pillars (Figure 6C). Thus, the recruitment of DAPK1 to adhesions was rapid but there was also a rapid release from soft pillars. To further determine if DAPK1 recruitment to or release from soft pillars was altered, we measured photobleaching recovery of EGFP-DAPK1 to soft and rigid pillars. The recovery rates were the same on both, indicating that binding was unaltered but release was accelerated on soft pillars (Supplementary Figures S4A,B). Thus, it seemed that soft matrices caused the rapid release of DAPK1 from adhesion sites, but recruitment continued.

### Death Associated Protein Kinase 1 Apoptotic Function is Altered by Src Phosphorylation and PTPN12

**FIGURE 7:**
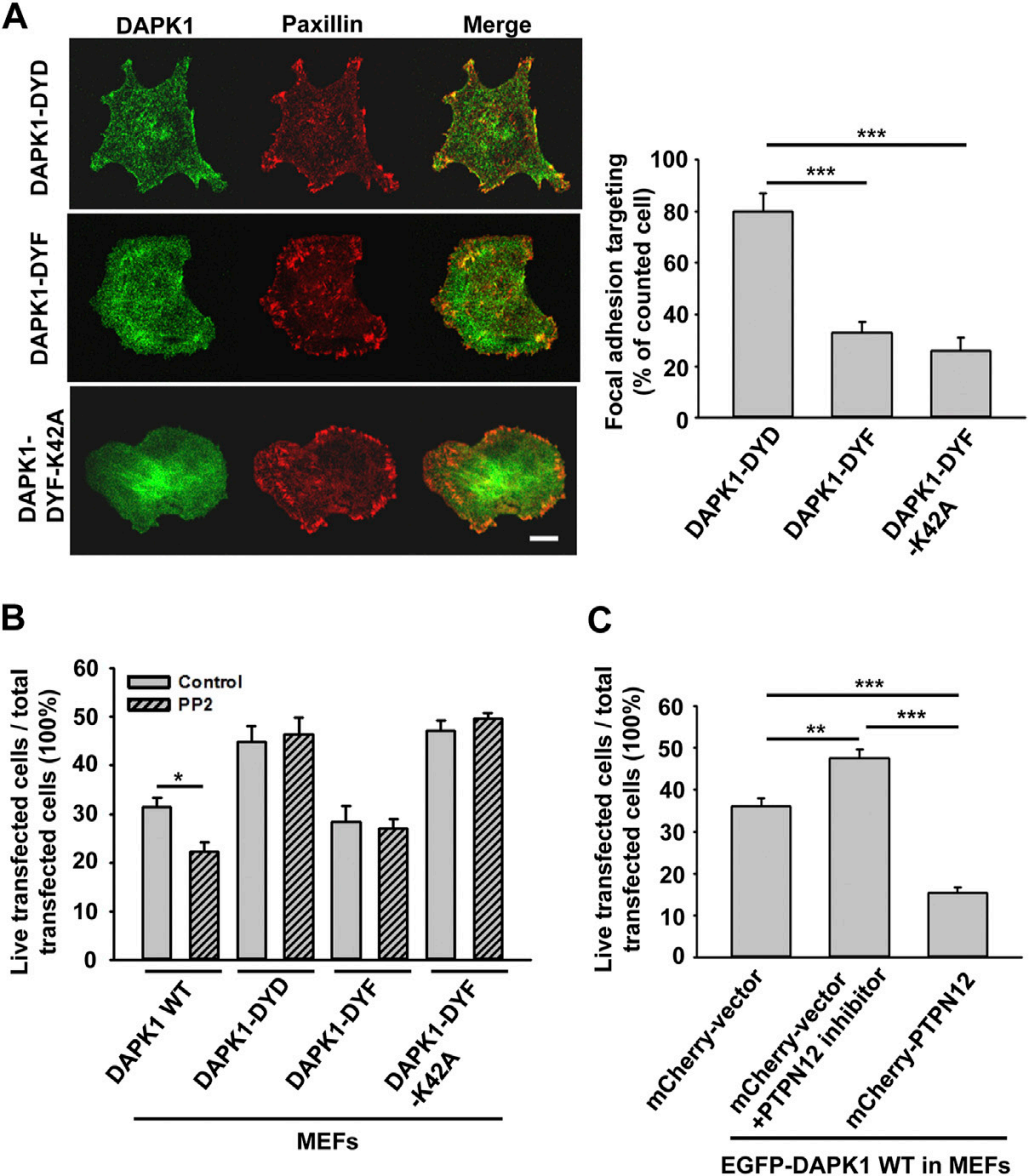
DAPK1 apoptotic activity is controlled by Src phosphorylation and PTPN12. **(A)** Left: Micrographs showing the distribution of DAPK1 in MEFs transfected with mCherry-paxillin and EGFP-DAPK1-DYD (phosphomimetic mutant), EGFP-DAPK1-DYF (non-phosphorylated mutant) or EGFP-DAPK1-DYF-K42A (non-phosphorylated inactive mutant) respectively after 30 min spreading on fibronectin-coated glass dishes. Scale bar: 10 μm. Right: Percentage of FA targeting positive cells were calculated from 200 transfected cells per group. ***p < 0.001. **(B)** MEFs were transfected with EGFP-DAPK1 WT, EGFP-DAPK1-DYD, EGFP-DAPK1-DYF or EGFP-DAPK1-DYF-K42A respectively for 1 day. The percentage of transfected cells that were still alive was quantified. The mean ± SEM of at least two independent experiments is described. For each experiment, 100–150 cells were analyzed for each transfection point. *p < 0.05, ***p < 0.001. **(C)** MEFs were transfected with EGFP-DAPK1 WT either co-transfected with mCherry-PTPN12 or treated with PTPN12 inhibitor. The percentage of co-transfected cells that were still alive was quantified. The mean ± SEM of at least two independent experiments is described. For each experiment, 100–150 cells were analyzed for each transfection point. **p < 0.01, ***p < 0.001.

In previous studies, tyrosine kinase/phosphatase activities were implicated in the regulation of DAPK1 (Wang et al., 2007; Maślikowski et al., 2017). To test if the vicinal tyrosines in DAPK1 (Y490/Y491) were involved, we overexpressed EGFP-DAPK1 mutants Y490D/Y491D (DYD, phosphomimetic mutant), Y490F/Y491F (DYF, non-phosphorylated mutant) or Y490F/Y491F-K42A (DYF-K42A, non-phosphorylated inactive mutant) in MEFs. Most of the Y-D mutation transfected cells (>80%) had concentrated DAPK1 in adhesion sites, whereas much less DAPK1 assembly in adhesion sites was observed in the DYF (∼30%) or DYF-K42A (∼20%) transfected cells (Figure 7A). In an apoptosis assay, the Y-F mutation enabled normal apoptosis whereas the Y-D mutation inhibited apoptosis, as did the Y-F double mutation of the inactive DAPK1-K42A (Figure 7B). Since Src is a key kinase that phosphorylates DAPK at vicinal tyrosines, leading to its inactivation (Wang et al., 2007), we used the Src family kinases (SFK) inhibitor PP2 and found that it caused a significant decrease in cell survival with wild type DAPK1, but did not affect the response of the cells expressing the DAPK1 tyrosine mutants (Figure 7B). Further, PP2 treatment led to inhibition of normal DAPK1 assembly into adhesions, as well as to a dramatic decrease in adhesion size (Supplementary Figures S5A,B). We next tested for apoptosis in the presence of Src siRNA and found an increase in DAPK1-induced cell death (Supplementary Figure S5C). Since the SFKs also activated EGFR in early spreading (Saxena et al., 2017a), we tested for the effect of EGFR inhibition on apoptosis and found enhanced cell death after EGFR inhibition (Supplementary Figure S5D). Thus, tyrosine phosphorylation of DAPK1 on the vicinal tyrosines enabled assembly of DAPK1 into adhesions and inhibited apoptosis, whereas the unphosphorylated DAPK1 could not assemble in adhesions but supported apoptosis.

**FIGURE 8:**
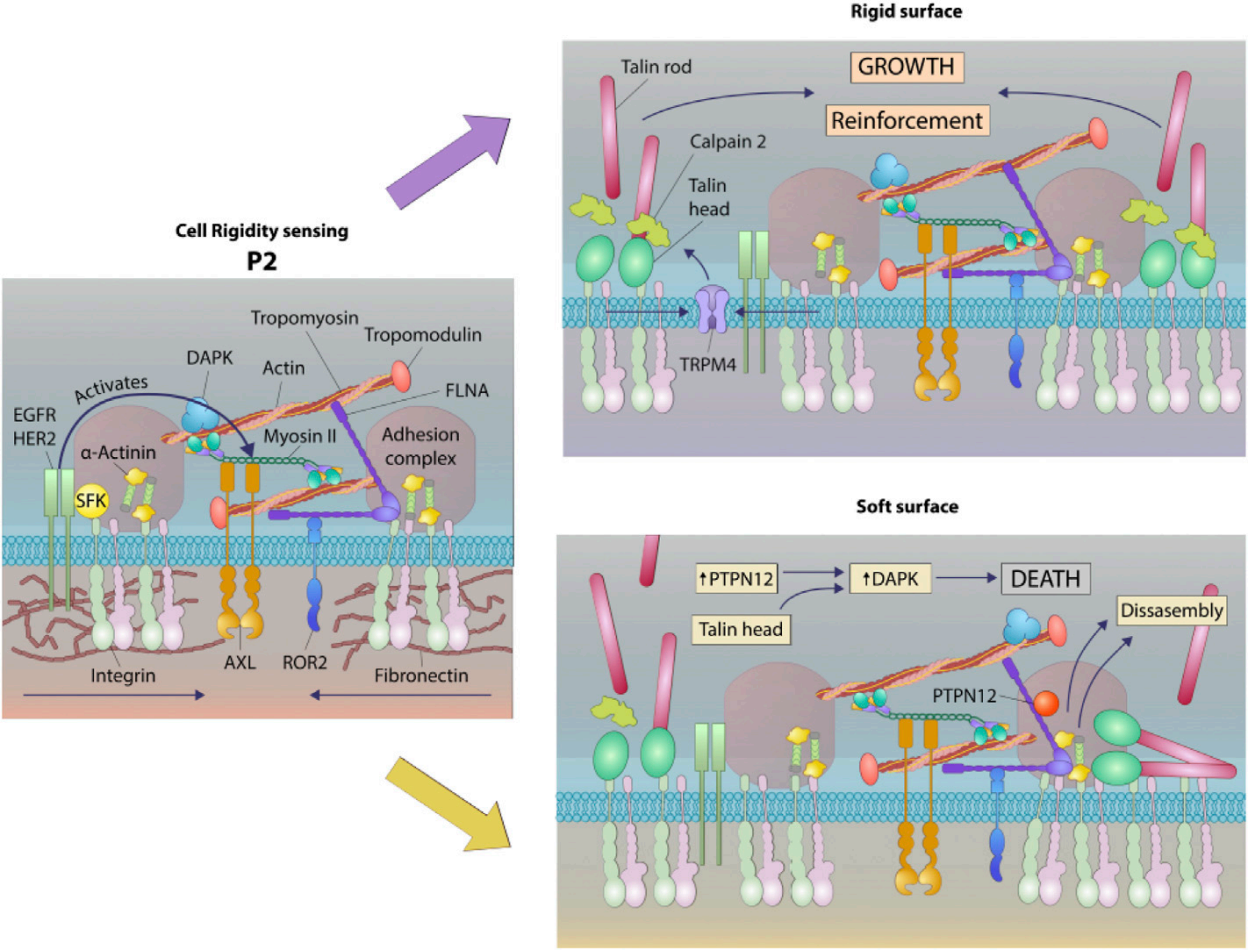
Schematic of a working model of DAPK1 shuttling depending on substrate rigidity. Rigidity sensing modules form upon early interaction with the matrix (Wolfenson et al., 2019). These modules include small integrin clusters that are connected at the intracellular side to actin fibers. Myosin II mini-filaments pull on the actin fibers from neighboring integrin clusters to a constant distance of ∼100 nm, leading to production of different force levels based on the external rigidity. High forces on stiff matrices lead to adhesion reinforcement through enhanced recruitment of proteins such as α-actinin, but on soft surfaces the forces that are developed are not high enough to induce reinforcement, leading eventually to adhesion disassembly (Wolfenson et al., 2016). These rigidity sensing modules are regulated at multiple levels, including by the receptor tyrosine kinases, AXL and ROR2, which regulate the displacement level and its duration, respectively (Yang et al., 2016). EGFR and HER2 also regulate the process by activating it on rigid but not on soft surfaces downstream of Src phosphorylation (Saxena et al., 2017a). The current study adds to this model the involvement of DAPK1, which has a dual role as a regulator of formation of mature adhesions on rigid matrices and in the activation of apoptosis on soft ones. In both cases, DAPK1 is recruited to the adhesions at early times through its interaction with Tpm1.1, that is, decorating the actin fibers and through its interaction with the talin head domain. The reinforcement of adhesions that occurs on stiff matrices leads to DAPK1 stabilization in the adhesions. If the surface is soft, then the phosphatase PTPN12 dephosphorylates DAPK1, leading to its activation to induce apoptosis as the adhesions disassemble.

Since DAPK1 assembled into adhesions on both soft and stiff matrices, we hypothesized that DAPK1 assembly into the adhesions was a normal part of the cell-matrix interaction and that a phosphatase was needed to disassemble adhesions on soft surfaces and to activate DAPK1 for apoptosis. A major tyrosine phosphatase that was implicated in cancer and motility was PTPN12 (Sastry et al., 2002; Li et al., 2015). To test for its possible involvement, we added an siRNA for and an inhibitor of PTPN12 and found that they blocked DAPK1 apoptotic function in MEFs (Figure 7C; Supplementary Figure S6A). Further, we observed an enhancement of apoptosis when we overexpressed PTPN12 (Figure 7C). In addition, we found that PTPN12 inhibitor increased inactive (p-308) DAPK1 staining at the edge of spreading cells (Supplementary Figure S6B). Thus, it seemed that tyrosine phosphorylation inhibited DAPK1 and apoptosis whereas PTPN12 activity was needed for activation of apoptosis on soft surfaces. These findings are summarized in a model diagram (Figure 8).

## Discussion

This study shows that DAPK1 has a major role both in the formation of matrix adhesions on rigid surfaces at early times and in the apoptosis of cells on soft surfaces. Both processes involve DAPK1 assembly in adhesions through its phosphorylation of Tpm1.1 at S283, as well as the proteolysis of talin1 to produce the head fragment. DAPK1 activity is needed for normal assembly of focal adhesions and proper rigidity sensing contractions. Further, the phosphorylation of DAPK1 on the vicinal tyrosine residues (Y490/491) by Src or EGFR is critical for its inactivation at adhesions. On sub-micrometer pillars with sub-cellular areas of different stiffness, the binding of DAPK1 to both soft and rigid pillars is initially similar, but once a maximal force is reached, rapid release is only found in the soft regions. DAPK1 activation, release, and adhesion disassembly all appear to correlate with the dephosphorylation of DAPK1 by PTPN12. Thus, the rigidity dependent activation of apoptosis involves DAPK1 activity in the formation of adhesions through Tpm1.1 and rigidity-dependent dephosphorylation of DAPK1. It is worth mentioning that ∼20% of MDA-MB-231 cells undergo anoikis in the absence of Tpm1.1 or in the presence of the non-phosphorylated mutant, S283A. Thus, at least some of the soft substrate induced cell death does not involve the DAPK1-Tpm1.1 axis. Further studies are needed to elucidate the additional pathway(s) involved. For example, the pro-apoptotic Bim protein, which can be activated in the absence of integrin dependent ERK activation, could be involved. Further, numerous pro-survival pathways can be activated when proper focal adhesions are formed, including FAK, integrin-linked kinase (ILK), Src, and PI3K (Taddei et al., 2012). Hence, such large regulatory networks could be inactivated, leading to cell death irrespective of DAPK1 activity.

An earlier study found that DAPK1 had a role in cell migration and polarization through an interaction with integrin and the talin1 head domain (Kuo et al., 2006). This is consistent with its function in the formation of contractile units and rigidity sensing; however, the longer-term reactions to rigid surfaces involve activation of dynamic responses beyond early rigidity sensing. Actin-dependent membrane extension correlates with rigidity sensing and fully spread cells have few rigidity-sensing contractions because they have few extensions (Saxena et al., 2017a). The cycle of mechano-testing and response can result in migration if there is polarization with a single leading edge. In the case of transformed cancer cells, there is a major change in cell state since they lack rigidity sensing contractions and Tpm1.1 in adhesions. It seems that normal rigidity sensing involves the recruitment of tyrosine phosphorylated DAPK1 along with Tpm1.1 to the contractile units. If the surface is rigid, then endogenous DAPK1 is stabilized in the adhesions along with Tpm1.1, talin1 head, and other focal adhesion proteins. If the surface is soft, then a phosphatase, such as PTPN12, dephosphorylates DAPK1 and causes its activation as well as the disassembly of the adhesions. Previous studies have shown that in immune cells the phosphatase LAR dephosphorylates DAPK1 (Wang et al., 2007); however, since the immune cells do not develop high forces on matrices, that may not be related to cell mechanics. The role of PTPN12 in activating *anoikis* is consistent with its role as a tumor suppressor (Li et al., 2015) and also its major effects on adhesion lifetime (Sastry et al., 2002).

The role of the tyrosine kinases in inactivating DAPK1 is consistent with previous studies that show high levels of phosphorylation of the vicinal tyrosine residues in cancer as well as fibroblast cells (Wang et al., 2007). In these studies, DAPK1 is inactivated upon phosphorylation by Src either directly or downstream through EGFR activation. In earlier studies, we found that Src activated EGFR or HER2 in cell spreading to catalyze rigidity sensing (Saxena et al., 2017a). Here we find that inhibitors of either will cause increased apoptosis, reinforcing the notion that DAPK1 is normally phosphorylated in the adhesions. Further, the inhibition of PTPN12 will block DAPK1 activation. On soft surfaces, there are few rigidity sensing contractions and few chances for assembly of DAPK1 into adhesion complexes with tyrosine phosphorylation (Saxena et al., 2017a).

It is logical that the activation of apoptosis on soft surfaces would involve engagement of DAPK1 with the rigidity sensing process at integrin adhesions. Rigidity sensing involves the assembly of sarcomere-like contractile units at integrin adhesion sites and the subsequent pulling of those adhesions to produce a total displacement of 100–120 nm. If the force developed is less than about 20–25 pN, then the matrix is considered soft and the adhesions disassemble, releasing activated DAPK1. Based upon these findings we hypothesize that Tpm1.1 and talin1 head domain form a complex with tyrosine phosphorylated DAPK1 that dissociates on soft surfaces due to the tyrosine phosphatase, PTPN12 (Figure 8).

The complex nature of the rigidity sensing module indicates that it integrates mechanosensing with several output signals depending upon the matrix rigidity. Although those output signals can vary dramatically for different cell types, the basic sensory machine appears to have many common elements. For example, the receptor-like-protein-tyrosine-phosphatase alpha (RPTPα) is needed for rigidity sensing in both fibroblasts and neurons but in hippocampal neurons the loss of rigidity sensing results in long straight neurites whereas in fibroblasts it results in transformed growth (Kostic et al., 2007). Since DAPK1 is part of rigidity sensing modules in fibroblasts, those cells can activate apoptosis on soft surfaces whereas DAPK1 is not linked to matrix rigidity when the sensory modules are missing cytoskeletal components and do not assemble as in cancer cells. Signaling from Src and RTKs phosphorylation is part of the rigidity-sensing module and is needed to inactivate DAPK1 as well as activate growth because of force-dependent, tyrosine phosphorylation on rigid substrates. The competing tyrosine phosphatases such as PTPN12 and possibly LAR are designed to keep cells from growing inappropriately when the matrix is soft but are not operative in the absence of the rigidity sensing modules. This raises many questions about how DAPK1 is inactivated when the rigidity sensors are missing and how are the phosphatases recruited to the adhesions on soft surfaces.

## Data Availability

The original contributions presented in the study are included in the article/Supplementary Materials, further inquiries can be directed to the corresponding authors.
