# Supplementary material for: Tumor Suppressor DAPK1 Catalyzes Adhesion Assembly on Rigid but Anoikis on Soft Matrices
Source: Front Cell Dev Biol. 2022 Jul 19;10:959521. doi: 10.3389/fcell.2022.959521 (PMC9343699; doi:10.3389/fcell.2022.959521)
Supplement: Supplementary file 1 [file DataSheet1.PDF]

Tumor suppressor DAPK1 catalyzes Adhesion Assembly on Rigid but Anoikis on Soft Matrices

Supplementary Material

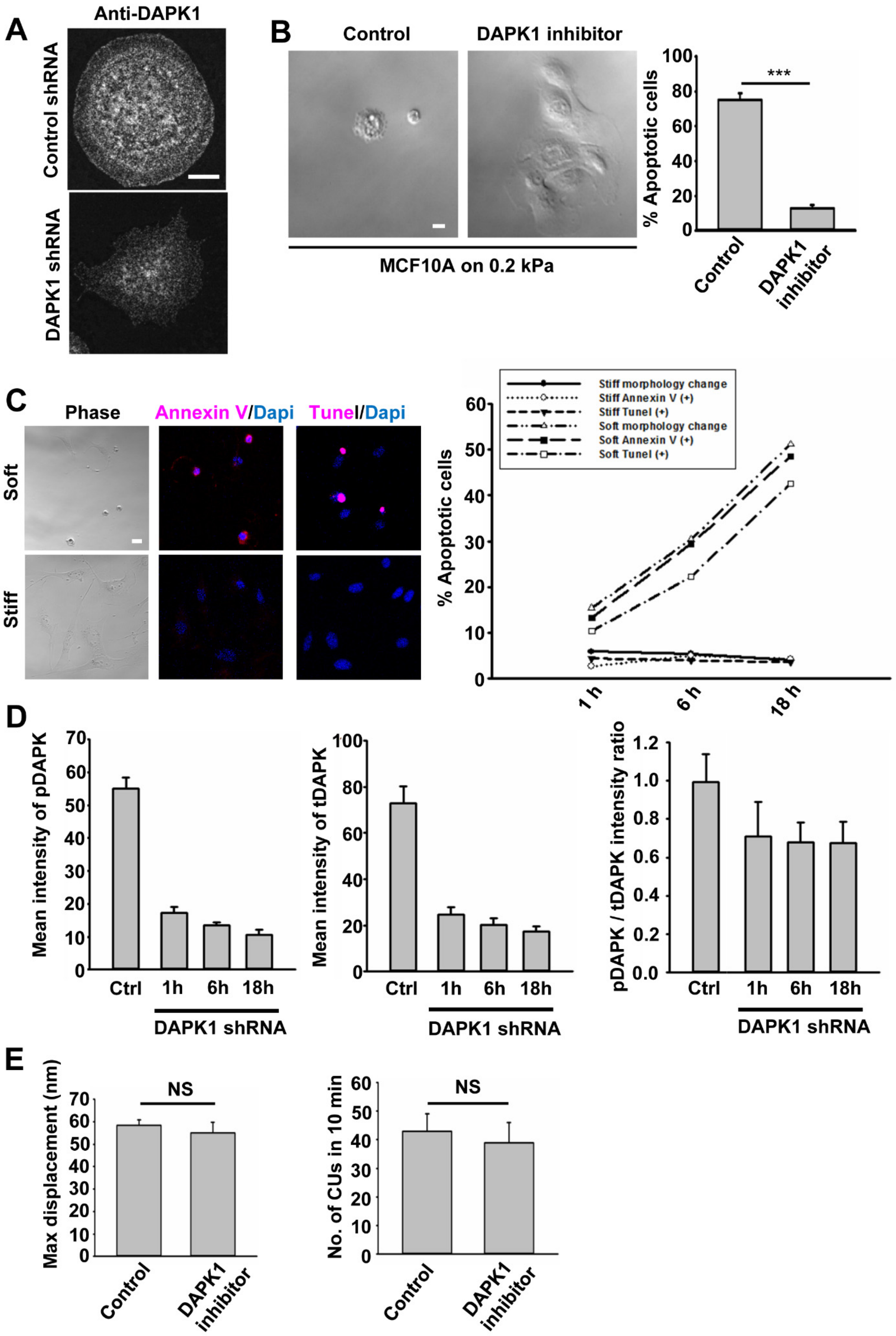
A
Anti-DAPK1
Control shRNA
DAPK1 shRNA
B
Control
DAPK1 inhibitor
MCF10A on 0.2 kPa
% Apoptotic cells
***
Control
DAPK1 inhibitor
C
Phase
Annexin V/Dapi
Tunel/Dapi
Soft
Stiff
Stiff morphology change
Stiff Annexin V (+)
Stiff Tunel (+)
Soft morphology change
Soft Annexin V (+)
Soft Tunel (+)
% Apoptotic cells
1 h
6 h
18 h
D
Mean intensity of pDAPK
Mean intensity of tDAPK
pDAPK / tDAPK intensity ratio
Ctrl
1h
6h
18h
DAPK1 shRNA
E
Max displacement (nm)
No. of CUs in 10 min
NS
Control
DAPK1 inhibitor

Supplementary Fig. 1 (**A**) MEFs transfected with control shRNA or DAPK1 shRNA were stained for anti-DAPK1 antibody. Scale bar: 10 μm. (**B**) DIC images of M10A cells with or without DAPK1 inhibitor after 2 days in culture on 0.2 kPa gels. Scale bar: 10 μm. Apoptotic cells were identified for appearance of apoptotic morphology including membrane blebbing and cell rounding. Graphs represent means ± SEM of at least two independent experiments. ***P <0.001. (**C**) MEF cells were incubated in culture for 1h, 6h or 18h on soft (5 kPa) or stiff gel surfaces (2000 kPa). Apoptotic cells were identified for appearance of apoptotic morphology including membrane blebbing and cell rounding. Live cells retained their normal spread morphology. Apoptotic cells were also determined by anti-Annexin V immunostaining (purple). The Life Technologies™ Click-iT® Plus TUNEL assay with Alexa Fluor® 647 dye (purple) was used to detect the fragmented DNA. Scale bar: 10 μm. The percentage of apoptotic cells was quantified. (**D**) DAPK1 shRNA MEFs (compare the intensity levels to MEF cells, Ctrl) were plated on 0.2 kPa gels. The levels of pS308 and total DAPK1 were monitored by double immunostaining of cells during over time. Graphs represent means ± SD. Experiment was repeated twice. ***P <0.001. (**E**) Means ± SEM of the maximal pillar displacements and the average number of CUs by MEFs before and after treatment with DAPK1 inhibitor. N > 30 pillars in each case.

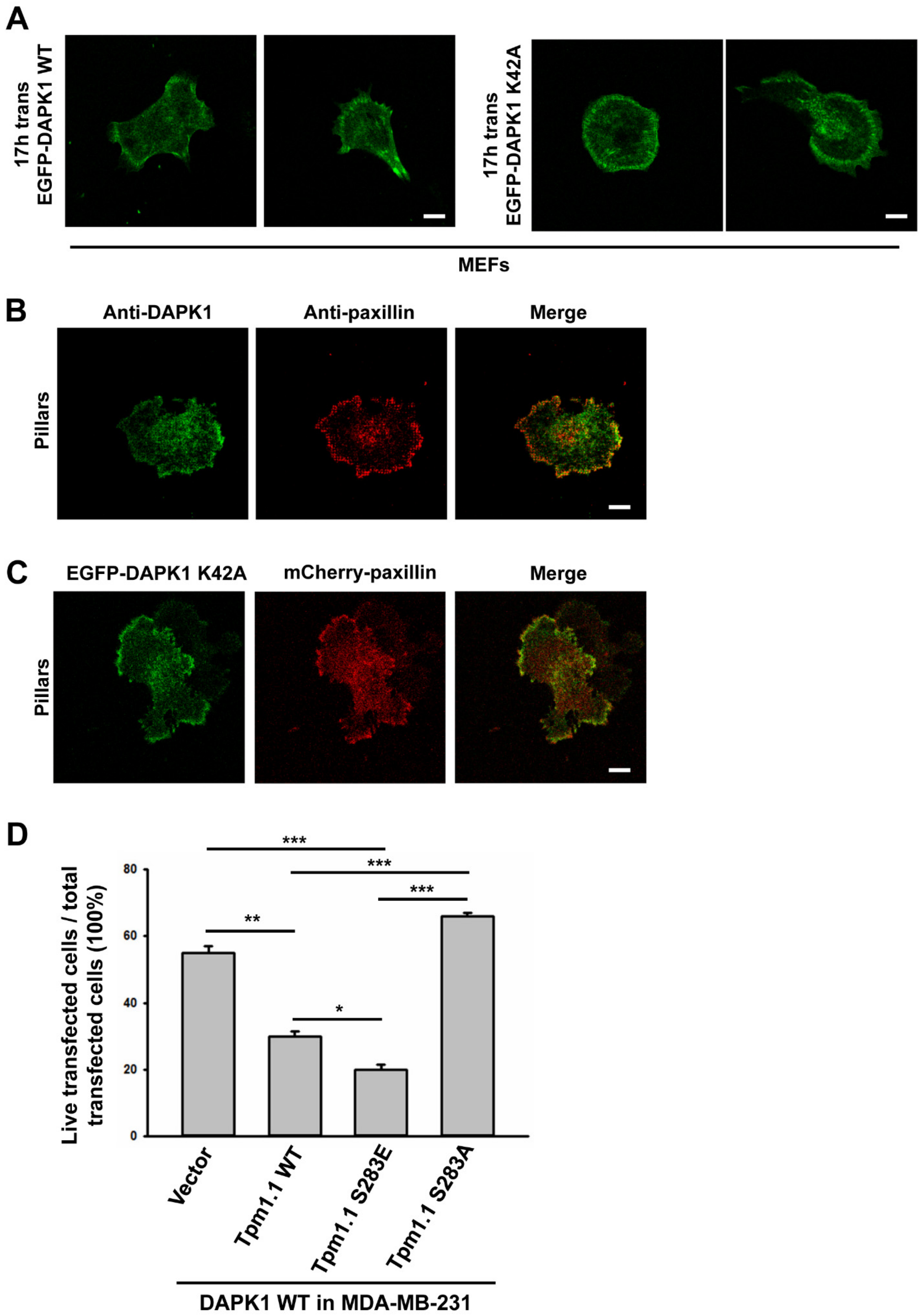
A
17h trans
EGFP-DAPK1 WT
17h trans
EGFP-DAPK1 K42A
MEFs
B
Anti-DAPK1
Anti-paxillin
Merge
Pillars
C
EGFP-DAPK1 K42A
mCherry-paxillin
Merge
Pillars
D
Live transfected cells / total transfected cells (100%)
0
20
40
60
80
***
***
***
**
*
Vector
Tpm1.1 WT
Tpm1.1 S283E
Tpm1.1 S283A
DAPK1 WT in MDA-MB-231

Supplementary Fig. 2 (**A**) Micrographs of MEFs transfected with EGFP-DAPK1 K42A or EGFP-DAPK1 WT for 17 hours on glass dishes. Scale bar: 10 μm. (**B**) MEFs were allowed to spread for 30 minutes on pillars (170 kPa), followed by anti-DAPK1/AlexaFluor 488 and anti-paxillin/AlexaFluor 555 immunostaining. Scale bar: 10 μm. (**C**) MEFs were co-transfected with EGFP-DAPK1 K42A and mCherry-paxillin. Cells were subsequently allowed to spread for 30 minutes on pillars (170 kPa). Scale bar: 10 μm. (**D**) MDA-MB-231 cells were co-transfected with DAPK1 WT and Tpm1.1 WT, Tpm1.1 S283E, Tpm1.1 S283A, or control vector on stiff dishes. The percentage of transfected cells that were alive was quantified. The mean ± SEM of at least two independent experiments is described. *P <0.05, **P <0.01, ***P <0.001.

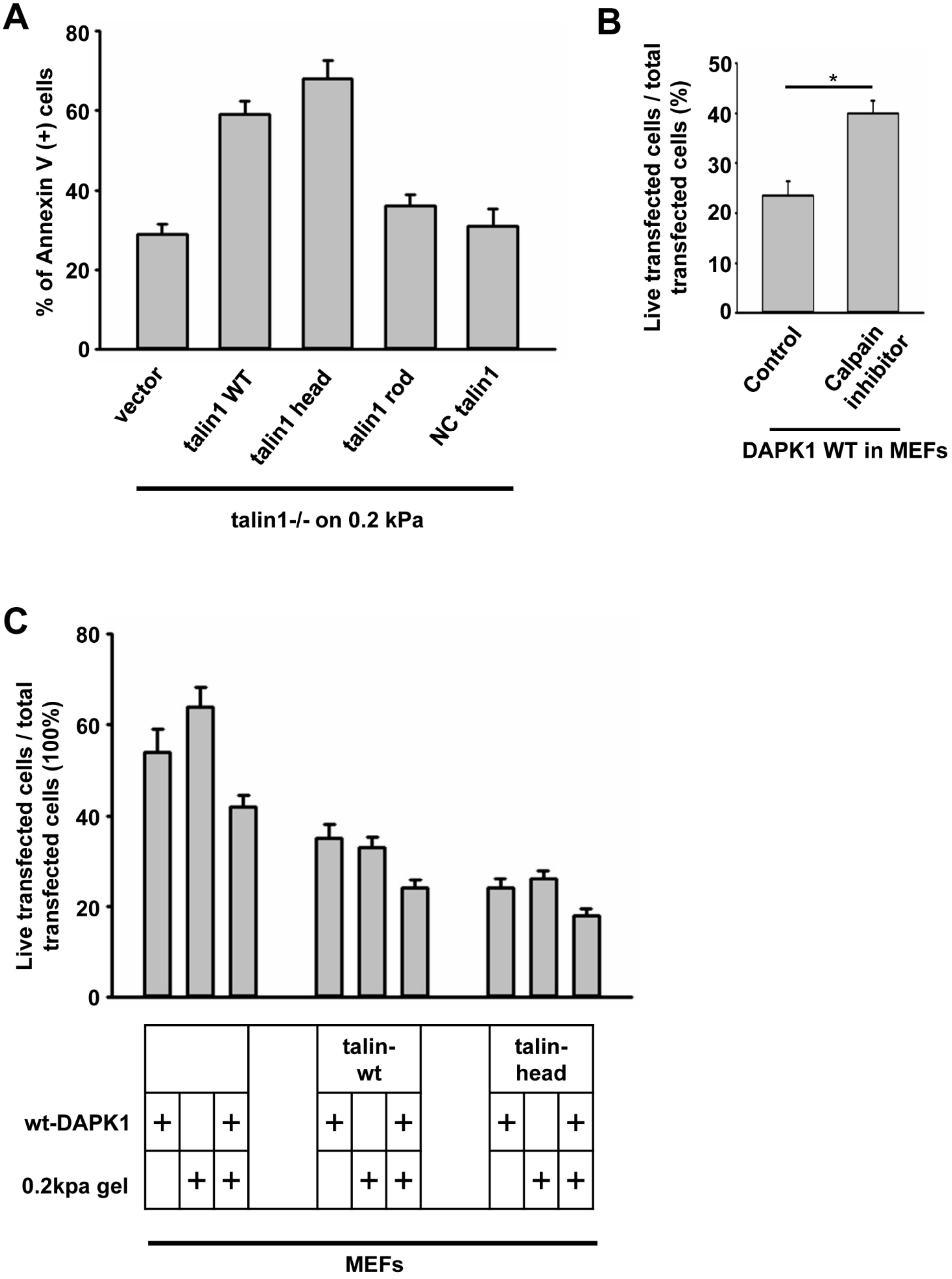
A
% of Annexin V (+) cells
0
20
40
60
80
vector
talin1 WT
talin1 head
talin1 rod
NC talin1
talin1-/- on 0.2 kPa
B
Live transfected cells / total transfected cells (%)
0
10
20
30
40
50
*
Control
Calpain inhibitor
DAPK1 WT in MEFs
C
Live transfected cells / total transfected cells (100%)
0
20
40
60
80
talin-wt
talin-head
wt-DAPK1
0.2kpa gel
MEFs

Supplementary Fig. 3 Talin1 head facilitates apoptosis on soft substrates. (**A**) Talin1$^{-/-}$ cells were transfected with talin constructs (talin1 WT, talin1 head, talin1 rod, non-cleavable talin1) or control vector and replated on fibronectin-coated 0.2 kPa gels for one day, followed by anti-Annexin V immunostaining. The percentage of Annexin V positive cells in transfected cells was quantified. The mean ± SEM of at least two independent experiments is described. For each experiment, 100-150 cells were analyzed for each transfection point. (**B**) MEFs transfected with EGFP-DAPK1 WT treated with DMSO or calpain inhibitor ALLN (100 μM). The percentage of transfected cells that were still alive was quantified. (mean ± SEM of ≥2 experiments with 100-150 cells; *P <0.05). (**C**) MEFs were co-transfected with DAPK1 WT and talin constructs (talin1 WT or talin1 head) and replated on fibronectin-coated 0.2 kPa gel dishes or glass dishes for one day. The percentage of transfected cells that were still alive was quantified (mean ± SEM of ≥2 experiments with 100~150 cells).

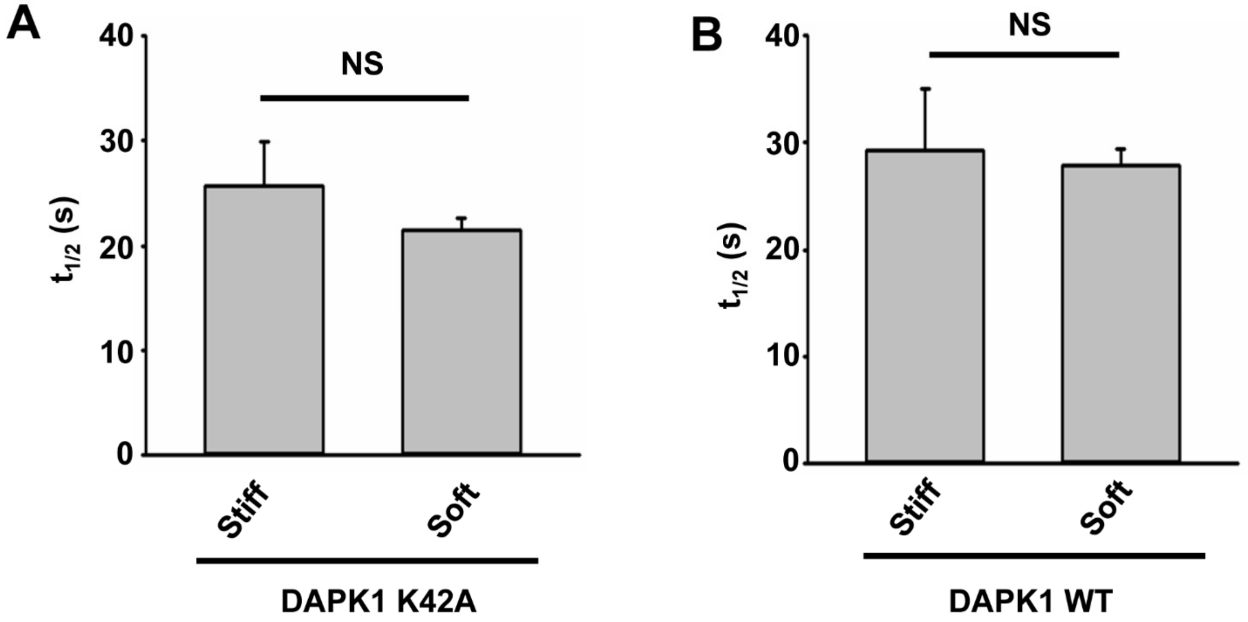

Supplementary Fig. 4 MEFs were transiently transfected with EGFP-DAPK1 K42A (**A**) or EGFP-DAPK1 WT (**B**) and allowed to spread on fibronectin-coated pillars for 20 minutes. Fluorescence recovery after photobleaching was measured using a confocal microscope. Graphs show half-times of fluorescence recovery on stiff (170 kPa) and soft (9 kPa) pillars. Values are means ± SEM. At least 5 different cells were analyzed in each group.

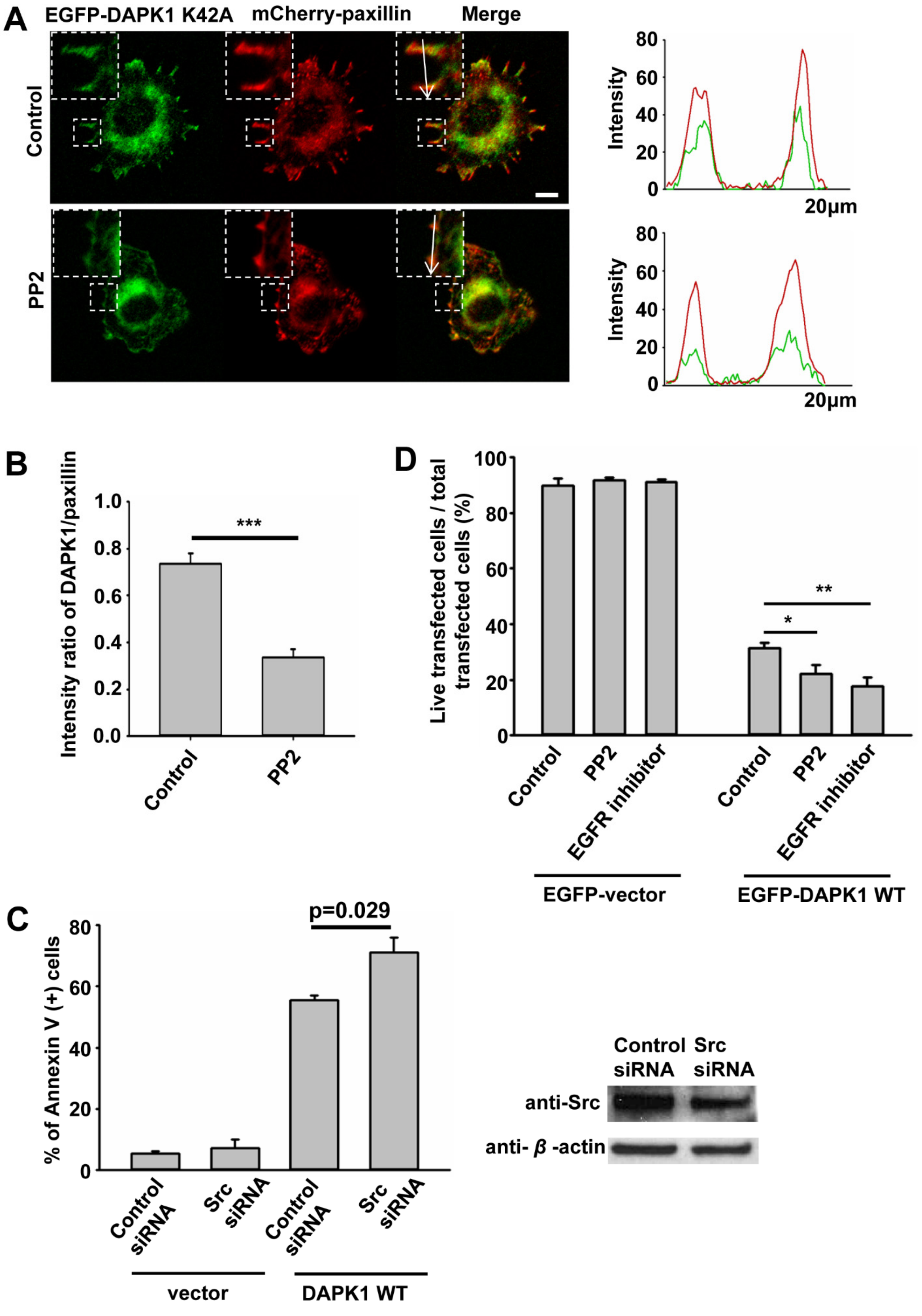
A
EGFP-DAPK1 K42A
mCherry-paxillin
Merge
Control
PP2
Intensity
20μm
B
Intensity ratio of DAPK1/paxillin
***
Control
PP2
C
% of Annexin V (+) cells
p=0.029
Control siRNA
Src siRNA
vector
DAPK1 WT
Control siRNA
Src siRNA
anti-Src
anti-β-actin
D
Live transfected cells / total transfected cells (%)
Control
PP2
EGFR inhibitor
EGFP-vector
EGFP-DAPK1 WT

Supplementary Fig. 5 (**A**) MEFs co-transfected with EGFP-DAPK1 K42A and mCherry-paxillin were pretreated with or without PP2 (200 nM) and replated on fibronectin-coated glass dishes for 30 minutes. The insets are the enlarged Epi microscope images of the boxed regions. Scale bar: 10 μm. Fluorescence intensity line profiles on the right are from the area of the representative adhesions covered by the white arrow in the merged images. Green line represents DAPK1. Red line represents paxillin. (**B**) The average intensity ratio of DAPK1 and paxillin in focal adhesions is presented. Graphs represent means ± SEM. ***P<0.001. N = at least 10 different cells in each group. (**C**) MEFs were transfected with EGFP-DAPK1 WT treated with either PP2 (200 nM) or EGFR inhibitor Gefitinib (10 nM) for one day. The percentage of live transfected cells per total transfected cells was quantified. Experiment was repeated three times. Graphs represent means ± SEM. *P <0.05. **P <0.01. (**D**) MEFs were transfected with scramble or Src siRNA for one day, followed by transfection with EGFP-DAPK1 WT for one day. Then cells were fixed, followed by anti-Annexin V immunostaining. The percentage of Annexin V positive cells in transfected cells was quantified. The mean ± SEM of at least two independent experiments is described. Western blot showing Src levels in MEFs treated with scramble or Src siRNA.

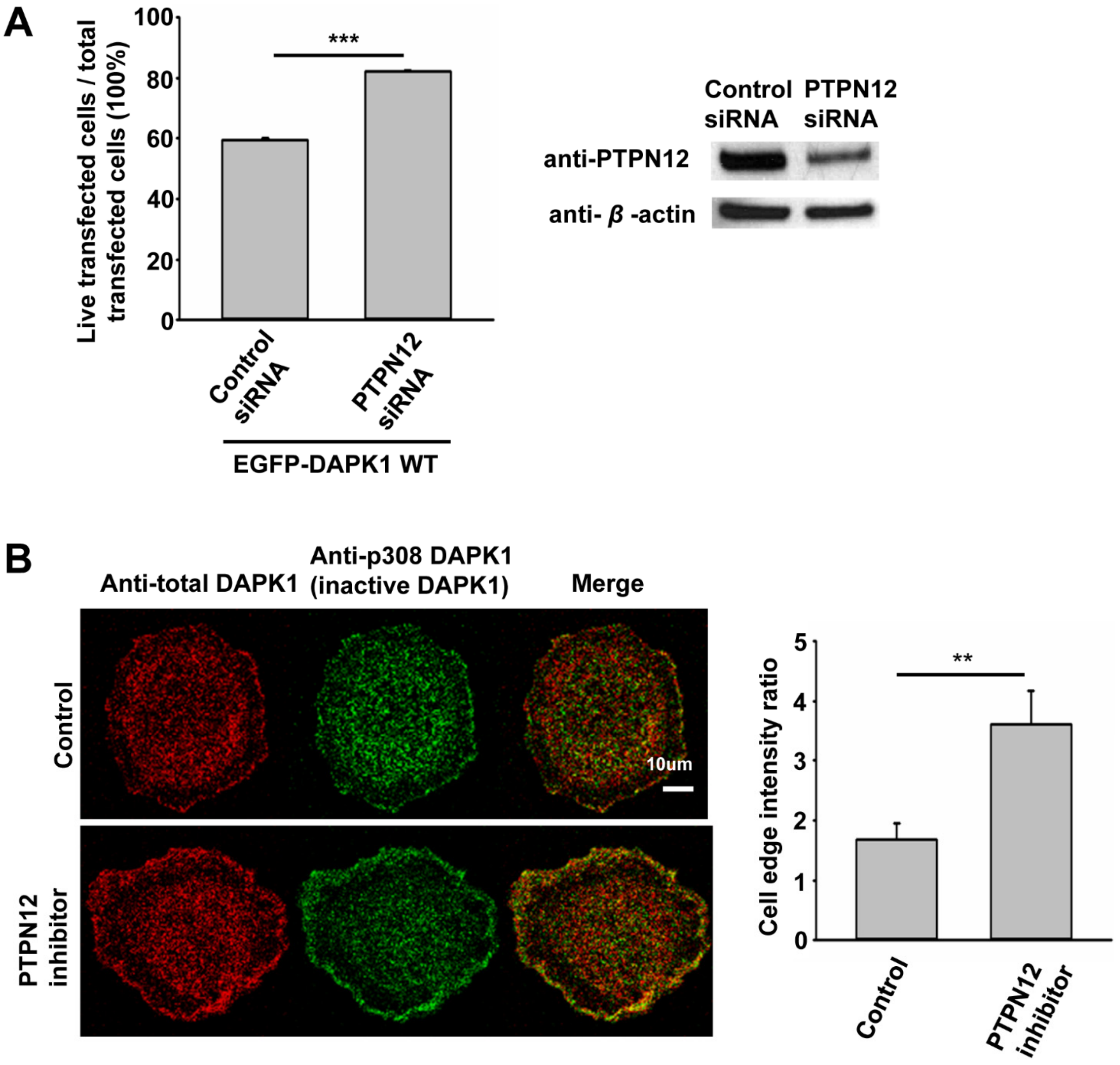

Supplementary Fig. 6 (**A**) MEFs were transfected with scramble or PTPN12 siRNA for one day, followed by transfection with EGFP-DAPK1 WT for one day. The percentage of live transfected cells per total transfected cells was quantified. The mean ± SEM of at least two independent experiments is described. ***P<0.001. Western blot showing PTPN12 levels in MEFs treated with scramble or PTPN12 siRNA. (**B**) MEFs treated with or without PTPN12 inhibitor were fixed after spreading on fibronectin-coated glass dishes for 30 minutes. Total DAPK1 and DAPK1-Ser308 phosphorylation was monitored by immunofluorescence. It shows that PTPN12 inhibitor increases inactive (p-308) DAPK1 staining at the edge of spreading cells. Scale bar: 10 μm.
